# The Effects of *Helicobacter pylori*-Derived Outer Membrane Vesicles on Hepatic Stellate Cell Activation and Liver Fibrosis In Vitro

**DOI:** 10.1155/2023/4848643

**Published:** 2023-04-12

**Authors:** Shahin Bolori, Saina Shegefti, Kaveh Baghaei, Abbas Yadegar, Kyung-Mee Moon, Leonard J. Foster, Mohammad Javad Nasiri, Hossein Dabiri

**Affiliations:** ^1^Microbiology Department, Faculty of Medicine, Shahid Beheshti University of Medical Sciences, Tehran, Iran; ^2^Gastroenterology and Liver Diseases Research Center, Research Institute for Gastroenterology and Liver Diseases, Shahid Beheshti University of Medical Sciences, Tehran, Iran; ^3^Basic and Molecular Epidemiology of Gastrointestinal Disorder Research Center, Research Institute for Gastroenterology and Liver Diseases, Shahid Beheshti University of Medical Sciences, Tehran, Iran; ^4^Foodborne and Waterborne Diseases Research Center, Research Institute for Gastroenterology and Liver Diseases, Shahid Beheshti University of Medical Sciences, Tehran, Iran; ^5^Department of Biochemistry & Molecular Biology, Michael Smith Laboratories, University of British Columbia, Canada

## Abstract

**Introduction:**

*Helicobacter pylori* is a prevalent pathogenic bacterium that resides in the human stomach. Outer membrane vesicles (OMVs) are known as nanosized cargos released by *H. pylori*, which have been proposed to have a key role in disease progression, pathogenesis, and modulation of the immune system. There are multiple evidences for the role of *H. pylori* in extragastroduodenal illnesses especially liver-related disorders. However, the precise mechanism of *H. pylori* extragastroduodenal pathogenesis still remains unclear. In the current study, we aimed to determine the impact of *H. pylori*-isolated OMVs on hepatic stellate cell (HSC) activation and expression of liver fibrosis markers.

**Materials and Methods:**

Five *H. pylori* clinical strains with different genotype profiles were used. *Helicobacter pylori* OMVs were isolated using ultracentrifugation and were analyzed by scanning electron microscopy (SEM) and dynamic light scattering (DLS). Liquid chromatography coupled with tandem mass spectrometry (LC-MS/MS) analysis was applied to determine protein components of *H. pylori*-derived OMVs. Cell viability of LX-2 human hepatic stellate cell line exposed to OMVs was measured by MTT assay. LX-2 cells were treated with OMVs for 24 h. The gene expression of *α*-SMA, E-cadherin, vimentin, snail, and *β*-catenin was analyzed using quantitative real-time PCR. The protein expression of *α*-SMA, as a well-studied profibrotic marker, was evaluated with immunocytochemistry.

**Results:**

Our results showed that *H. pylori* strains released round shape nanovesicles ranging from 50 to 500 nm. Totally, 112 various proteins were identified in OMVs by proteomic analysis. The isolated OMVs were negative for both CagA and VacA virulence factors. Treatment of HSCs with *H. pylori*-derived OMVs significantly increased the expression of fibrosis markers.

**Conclusions:**

In conclusion, the present study demonstrated that *H. pylori*-derived OMVs could promote HSC activation and induce the expression of hepatic fibrosis markers. Further research is required to elucidate the definite role of *H. pylori*-derived OMVs in liver fibrosis and liver-associated disorders.

## 1. Introduction

*Helicobacter pylori* is a well-studied Gram-negative, microaerophilic, and motile pathogenic microorganism that is able to colonize the human stomach [1]. *Helicobacter pylori* strains can express various virulence factors such as urease, flagellum, BabA, SabA, OipA, HopQ, CagA, VacA, and HtrA, which facilitate its attachment, colonization, and disease development. Recently, *H. pylori*-derived outer membrane vesicles (OMVs) have been proposed as a potent virulence marker involved in the pathogenesis of this bacterium [2]. OMVs are nanosized structures released from live bacteria in a logarithmic phase of growth and carry a lot of factors such as enzymes, phospholipids, nucleic acids, toxins, and any different proteins rooted from cytoplasm, membrane, and outer membrane [3]. These extracellular vesicles (EVs) can be engulfed by different eukaryote cells via different endocytosis strategies and transfer several virulence factors directly into the host cell cytoplasm [4, 5]. Previous investigations have demonstrated that *H. pylori*-derived OMVs can contribute to disease development, and it is documented that OMVs promote local and systemic inflammatory responses and cause gastric barrier dysfunction [2, 3].

Traditionally, *H. pylori* is known as the major causative agent for developing various gastroduodenal pathologies including peptic ulcer disease, gastritis, gastric adenocarcinoma, and mucosal-associated lymphoid tissue (MALT) cancer [6]. Nowadays, there are several reports for the role of *H. pylori* in extragastroduodenal diseases such as neurodegenerative disease, inflammatory bowel disease (IBD), celiac disease, and also liver-related disorders [7]. Also, several studies have documented a positive association between nonalcoholic fatty liver disease (NAFLD) and *H. pylori* infection [8, 9]. However, any attempt targeting *H. pylori* detection or culture from liver and hepatic tissues has failed so far [10].

Liver fibrosis is a cell response in liver chronic inflammation that may lead to hepatocellular carcinoma (HCC) under uncontrolled conditions. Viral or autoimmune hepatitis, alcohol abuse, NAFLD, and microbial infections are the main causes of liver inflammation [11]. Liver inflammation can result in activation of hepatic stellate cells (HSCs), which are the key players mediating liver fibrosis [12]. Activation of HSCs requires the change from a quiescent to a proliferative, fibrogenic, and migratory phenotype (i.e., myofibroblast), which is characteristic of hepatic fibrogenesis process [13].

So far, little is known regarding the role of OMVs released from *H. pylori* clinical strains and their cargo in development of extragastroduodenal diseases in particular liver-associated disorders. In the present study, we aimed to investigate the direct effects of *H. pylori*-derived OMVs on HSCs activation, and their capability to induce liver fibrosis markers using LX-2 cell line.

## 2. Materials and Methods

### 2.1. *H. pylori* Strains and Culture

A collection of five different clinical strains of *H. pylori* obtained from nonrepetitive patients in our previous works were used in this study [14, 15]. The clinical and genotypic characteristics of selected *H. pylori* strains are shown in Table 1. Brucella agar (Merck, Darmstadt, Germany) enriched with 10% fetal calf serum (FCS), 7% (*v*/*v*) horse blood, and Skirrow supplement (polymyxin 0.05 mg/L, vancomycin 2.0 mg/L, and trimethoprim 1.0 mg/L) was used to culture the strains. The culture plates were kept at 37°C in microaerophilic conditions for 3-5 days. *Helicobacter pylori* strains were confirmed by Gram stain technique, colony morphology, and positive reactions to biochemical tests such as catalase, oxidase, and urease. Pure cultures from the selected strains were stored in brain heart infusion medium (Merck, Darmstadt, Germany) enriched with 20% FCS and 15% glycerol at -80°C.

### 2.2. OMV Isolation and Characterization

To isolate OMVs, *H. pylori* strains were cultured in Brucella broth medium (Merck, Darmstadt, Germany) enriched with 10% FCS for 72 h at 37°C under microaerobic conditions with continuous rotation (120 rpm). Then, supernatants were concentrated using centrifugations (10,000 × g, 20 min and 4°C).

After centrifugations, supernatants were filtered through a 0.45 *μ*m cellulose filter membrane (MCE, USA), and OMVs were extracted using ultracentrifugation (Optima XE-100; Beckman Coulter, USA) at 200,000 × g, for 3 h at 4°C as previously described with slight modifications [16]. The isolated OMVs were resuspended in sterile PBS and frozen at -80°C until use. Dynamic light scattering (DLS) and scanning electron microscopy (SEM) were performed to identify the OMVs size and morphology. BCA assay and sodium dodecyl sulphate-polyacrylamide gel electrophoresis (SDS-PAGE) were used to determine the protein concentration and content of OMVs.

### 2.3. OMVs Proteome

For OMV proteomics, in the first step, samples were heated at 99°C for 5 min in SDS sample buffer to stabilize them and eliminate any protease activity. For SDS-PAGE, samples were subjected to electrophoresis in SDS-10% polyacrylamide gel electrophoresis based on the previous published method [17]. The gels were stained with Coomassie blue for visualizing the proteins bands. For each sample, the entire lane of stained protein was cut from the gel and digested with trypsin overnight as previously published [18]. Resulting peptides were desalted with STAGE tips based on previously described protocols [19]. After peptide preparation, 2 *μ*g of each sample was injected into quadrupole-time of flight mass spectrometry (Impact II, Bruker Daltonics) coupled to an EASY-nLC 1000 (Thermo Fisher Scientific, USA) with previously described parameters [20]. Final results were analyzed by MaxQuant version 1.6.7.0 [21] and searched against *H. pylori* databases from the Uniprot database.

### 2.4. Cell Culture and Viability Assay

To examine the effects of OMVs on HSC activation, LX-2 cells were treated with the representative OMVs obtained from *H. pylori* clinical strain no. 1 as indicated in Table 1. The LX-2 human hepatic stellate cell line was kindly provided by Professor Friedman (Mount Sinai School of Medicine, New York, USA). LX-2 cells were cultured in complete Dulbecco's modified Eagle's medium (DMEM) enriched with 2% (*v*/*v*) heat-inactivated fetal bovine serum (FBS) (Gibco/Invitrogen, Carlsbad, CA, USA), 100 U/mL of penicillin, 100 *μ*g/mL of streptomycin, and 2 mM of L-glutamine. To determine the LX-2 cells viability, MTT assay was carried out with the commercial Cell Proliferation Kit I (Sigma-Aldrich, St. Louis, Missouri, USA) based on the producer's protocol. In brief, LX-2 cells at a density of 5 × 10^3^ cells per each well were seeded in 96-well plates and then separately contaminated and incubated with varying concentrations (1, 5, 10, 15, 20, and 25 *μ*m/mL) of *H. pylori* OMVs for 24 h. At the indicated time point, 10 *μ*L of MTT reagent (3-(4,5-dimethylthiazol-2-yl)-2,5-diphenyltetrazolium bromide) was added to each well, and the cells were kept for 4 h at 37°C. The process was stopped with dimethyl sulfoxide (DMSO), color developing substance, and absorbance at 560 nm was documented using a microplate reader (ELx808, BioTek Instruments, Winooski, Vermont, USA). The percentage of LX-2 cell viability was determined using the formula: cell viability (%) = (absorbance of treated cells × 100%)/absorbance of untreated cells.

### 2.5. LX-2 Cell Treatments

LX-2 cells were cultured in 6-well plates at a density of 5 × 10^5^ cells in each well and kept at 37°C for 24 h in a CO_2_ incubator. Before treatments, the seeded cells were washed with PBS (pH 7.2), and the culture media were changed with DMEM medium. After that, the LX-2 cells were contaminated with 5 *μ*g/mL of *H. pylori*-derived OMVs for 24 h. In addition, the untreated LX-2 cells were used as the control group. The experiments were performed in triplicate and repeated at least three times. After 24 h, the cell pellets were used for RNA extraction and gene expression assays.

### 2.6. Immunocytochemistry

The protein expression of *α*-SMA, as a well-studied profibrotic marker for the evaluation of HSC activation and fibrosis, was determined in the OMV-treated LX-2 cells by using immunocytochemistry (ICC) technique. Briefly, cells were seeded into six-well plates at a density of 5 × 10^5^ cells/well, exposed to *H. pylori* OMVs at concentration of 5 *μ*g/mL for 24 h. Treated cells were washed with sterile PBS (pH 7.2) for three times, fixed with 4% paraformaldehyde in sterile PBS (pH 7.5), and their membrane permeabilized using 0.5% Tween-20 in 5% bovine serum albumin (BSA) blocking solution for 30 min. For analysis of *α*-SMA, cells were incubated with rabbit polyclonal *α*-SMA antibody (1 : 100, Cell Signaling, Cat#: GTX10034) 24 h at 4°C. After 24 h, the treated cells were incubated with a goat anti-rabbit IgG Alexa Fluor 488 (Life Technologies, Cat# BA1054-2) for 1 h at ambient temperature. Also, DAPI staining was used for 15 min to see the fixed cells nuclei. Fluorescent images were documented by the Cytell-TM Cell Imaging System (GE, Healthcare, UK). The protein expression level was reported as the fold change comparing to the untreated control cells as negative control.

### 2.7. RNA Extraction and cDNA Synthesis

Total RNA was extracted from OMV-treated cells and untreated LX-2 cells, with the FavorPrep™ blood and cell total RNA mini kit (Favorgen® Biotech Corp., Pingtung, Taiwan) based on the developer's instruction. The RNA samples were stored at -80°C for further gene expression analysis. The quality and concentration of extracted RNAs were determined by measuring absorbance at 260 nm with a NanoDrop® ND-1000 spectrophotometer (Thermo Scientific, USA). RNAs were reverse transcribed to cDNA using the commercial cDNA synthesis kit (Favorgen® Biotech Corp., Pingtung, Taiwan) based on the manufacturer's instruction.

### 2.8. Quantitative Real-Time PCR

The quantitative real-time PCR assay was performed to investigate *β*-catenin, E-cadherin, snail, vimentin, and *α*-SMA genes expression level using the Rotor-Gene® Q real-time PCR system (Qiagen, Germany) and BioFACT™ 2X Real-Time PCR master mix (Biofact, Daejeon, South Korea). The primers and the size of each product used to examine the expression of *β*-catenin, E-cadherin, snail, vimentin, and *α*-SMA genes are presented in Table S1. The GAPDH housekeeping gene was used as the endogenous control. To evaluate the amplification process, melting curve analysis was used after each run. All reactions were run for three times. The relative gene expression was determined by applying the 2^-*ΔΔ*Ct^ method, and the mRNA expression level was given as the fold change comparing to the untreated control cells.

### 2.9. Statistical Analysis

The obtained data were analyzed using the GraphPad Prism 8 (GraphPad Software, Inc., USA). One-way ANOVA was applied to calculate variations among different groups, and comparisons between each group were analyzed using *t*-test. Results were shown as the average ± standard error of the mean (SEM) of at least triplicate experiments, unless otherwise stated. Differences among the study groups were considered statistically significant when *P* < 0.05, ^∗^*P* < 0.05, ^∗∗^*P* < 0.01, ^∗∗∗^*P* < 0.001, and ^∗∗∗∗^*P* < 0.0001.

## 3. Results

### 3.1. Characterization of *H. pylori* OMVs and Their Protein Contents

**Figure 1:**
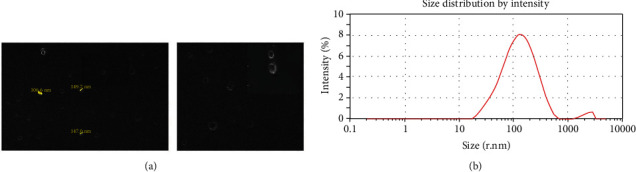
Morphological characterization of *H. pylori*-derived OMVs. (a) Scanning electron microscopic (SEM) image of OMVs indicated the round-shaped and double-layered vesicles in various sizes (range: 50-200 nm). (b) Size distribution by intensity of *H. pylori*-derived OMVs based on DLS. Size distribution is determined based on the intensity of OMVs in the ultracentrifugation technique. DLS confirmed nanosized OMVs in a range of about 50 to 450 nm, peaked at 100-150 nm.

The cell-free OMVs extracted from *H. pylori* clinical strains were purified by ultracentrifugation. The phenotypic characterization of OMVs was evaluated using DLS and SEM, respectively. Obtained data revealed that OMVs isolated from *H. pylori* clinical strains are round shape with a bilayer membrane and were ∼50-450 nm in diameter (Figure 1). This showed that OMVs were purified successfully without contamination with other bacterial components for subsequent cell experiments. The SDS-PAGE revealed protein bands with varying molecular weights, from 11 to 245 kDa, which shows the protein content of OMVs. To explore the protein contents of *H. pylori*-derived OMVs, liquid chromatography coupled with tandem mass spectrometry (LC-MS/MS) analysis was applied, and 112 different proteins were identified. Proteins are categorized in different classes including the following: 9 (8%) outer membrane proteins, 7 (6.2%) protein with unknown functions, 2 (1.7%) antibiotic resistance-associated proteins, 10 (8.9%) colonization factors, 5 (4.5%) acid resistance-related proteins, 12 (10.7%) potent virulence factors, 50 (44.6%) metabolism and vital functions, 3 (2.6%) motility, 3 (2.6%) ribosomal proteins, 2 (1.7%) iron-related proteins, and 11 (9.8%) others, ranked by abundance in Table 2. Among these different proteins that were detected, 47 (41.9%) proteins were common in the OMVs derived from *H. pylori* strains numbers 1, 2, 4, and 5 (OMV 1 had 8 (7.1%) unique proteins, OMV 2 had 13 (11.6%) unique proteins, OMV 4 had 30 (26.7%) unique proteins, and OMV 5 had 17 (15.1%) unique proteins) as indicated in Table S2. In addition, there were no unique proteins identified in OMV 3, which was obtained from a less virulent *H. pylori* strain. Among various proteins, urease and catalase enzymes were abundant, and metabolism plus proteins with vital functions was more condense. Also, all extracted OMVs were negative for both CagA and VacA virulence proteins.

### 3.2. Effects of *H. pylori*-Derived OMVs on LX-2 Cell Viability

**Figure 2:**
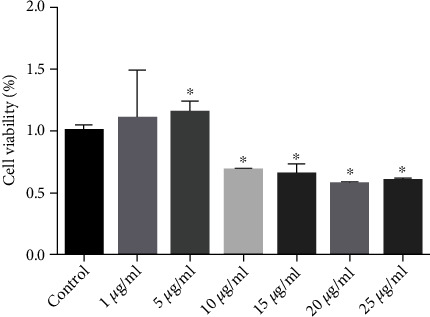
Effects of varying concentrations of the representative OMVs obtained from *H. pylori* clinical strain no. 1 (1, 5, 10, 15, 20, and 25 *μ*g/mL) on cell viability of the LX-2 cells. Data are shown as the mean ± SEM. ^∗^*P* < 0.05.

To assess cytotoxic effects of OMVs on HSCs, direct microscopic examination and cell viability assay were performed. Compared with the control cells after 24 h, OMVs derived from *H. pylori* induced significant increase in the number of viable LX-2 cells with 1 and 5 *μ*g/mL concentrations during the coculture time. Furthermore, MTT assay indicated that *H. pylori* OMVs at concentrations of 10, 15, 20, and 25 *μ*g/mL significantly affected the number of LX-2 cells in contrast to untreated control cells (Figure 2). Accordingly, OMVs with 5 *μ*g/mL concentration was used in further coculture assays with LX-2 cells.

### 3.3. OMVs Promoted Gene Expression of Fibrogenesis Markers in LX-2 Cells

**Figure 3:**
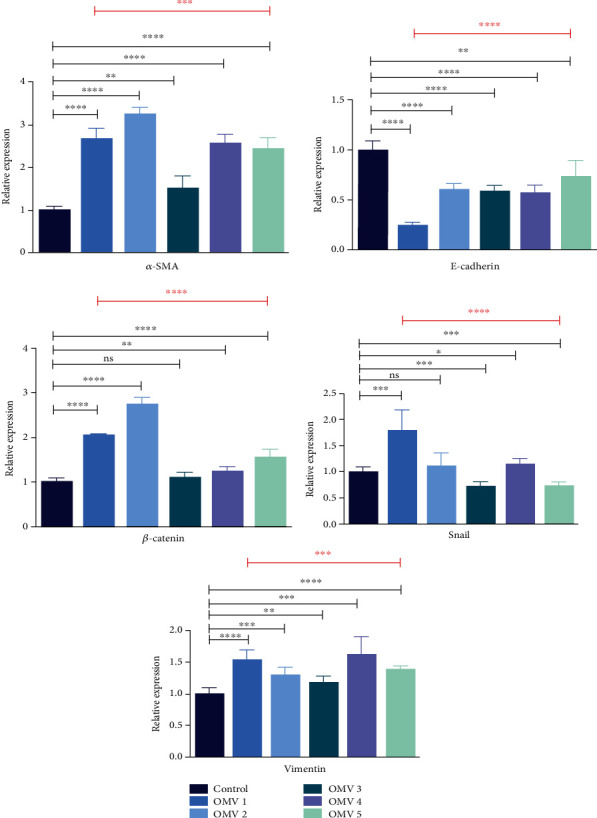
Gene expression of liver fibrosis markers in LX-2 cells upon treatment with 5 *μ*g/mL of *H. pylori* OMVs. Relative gene expression of fibrosis markers (*α*-SMA, *β*-catenin, and vimentin) was markedly increased after treatment of LX-2 cells with OMVs derived from different *H. pylori* strains. The mRNA level of E-cadherin was decreased in LX-2 cells treated with OMVs isolated from different *H. pylori* strains. However, OMVs isolated from *H. pylori* strains numbers 1, 2, and 4 upregulated the expression of snail, while OMVs derived from strains numbers 3 and 5 decreased its expression in comparison with untreated cells. Data presented as means ± standard error (SEM) for three independent experiments. Data are shown as the mean ± SEM. ^∗^*P* < 0.05, ^∗∗^*P* < 0.01, ^∗∗∗^*P* < 0.001, and ^∗∗∗∗^*P* < 0.0001 by post hoc one-way ANOVA statistical analysis.

To evaluate the expression of fibrotic markers in stimulated LX-2 cells, qRT-PCR was performed to detect the mRNA expression levels of E-cadherin, vimentin, *β*-catenin, *α*-SMA, and snail. The GAPDH gene was used as a reference gene. As shown in Figure 3, OMVs isolated from *H. pylori* strains numbers 1, 2, 4, and 5 significantly elevated the gene expression of *α*-SMA, *β*-catenin, and vimentin expression level in comparison to control cells. Moreover, OMVs derived from different *H. pylori* strains applied in this work downregulated the expression level of E-cadherin as compared to control cells. Regarding the snail expression, OMVs isolated from *H. pylori* strains numbers 1, 2, and 4 upregulated the expression of this marker, while OMVs derived from strains numbers 3 and 5 decreased its expression in comparison with untreated cells. These results demonstrate the capability of *H. pylori* OMVs to activate HSCs and stimulate the expression of liver fibrosis markers. Furthermore, there was no significant difference in the ability of OMVs originated from *H. pylori* strain number 3 (as less virulent strain according to genotyping results) to modulate the gene expression level of fibrosis markers, except for *β*-catenin, in comparison to OMVs derived from other *H. pylori* strains.

### 3.4. OMVs Increased the Protein Expression of *α*-SMA in LX-2 Cells

**Figure 4:**
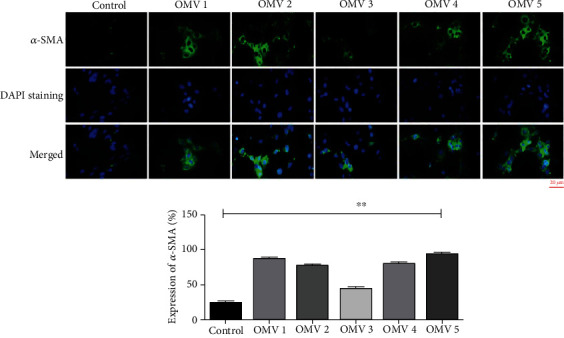
Protein expression of *α*-SMA in LX-2 cells upon treatment with 5 *μ*g/mL of *H. pylori* OMVs. Relative protein expression of *α*-SMA was markedly increased after treatment of LX-2 cells with 5 *μ*g/mL of *H. pylori* OMVs. Data presented as means ± standard error (SEM) for three independent experiments. Statistical significance was determined using one-way ANOVA with ^∗∗^*P* < 0.01. Scale bar: 20 *μ*m, magnification 200X.

Immunocytochemistry was used to measure the protein expression level of *α*-SMA, as an important marker, which is overexpressed during HSC activation. Protein expression of *α*-SMA mostly confirmed the gene expression in the different studied groups. Our findings suggest that *H. pylori* OMVs have noticeable potential to increase more than twofold of the expression of HSC fibrotic markers as compared with untreated HSCs that served as control cells (Figure 4).

## 4. Discussion

*Helicobacter pylori* is a well-known and important pathogen involved in different gastric diseases such as acute and chronic gastritis, gastric atrophy, and gastric malignancy [22]. It is clear that *H. pylori* infection can lead to serious diseases in the stomach and duodenum, but there has also been growing documents of its clinical consequences in extragastroduodenal diseases. According to recent studies, *H. pylori* infection has been related to different disorders in the gastrointestinal tract, and there has been hypothesis suggesting possible association of *H. pylori* infection with NAFLD development, which is the well-studied hepatic syndrome associated with chronic disorders such as obesity, diabetes, and cardiovascular disease [23]. The evidence for *H. pylori* and NAFLD association focused on the detection of anti-*H. pylori* IgG in patients suffering from NAFLD [24–27]. Liver fibrosis is a multifactorial and complex process especially because of HSC activation and high overexpression of liver fibrosis markers which can cause hepatocellular dysfunction [26]. The principal extracellular causative agents that cause liver fibrosis include chronic infections and epithelial cell damage. To the author's knowledge, until now, *H. pylori* culture has been negative among liver biopsies. However, in some studies *H. pylori* DNA was detected in liver, which could be likely due to the presence of DNA in *H. pylori*-derived OMVs [28, 29]. These observations highlighted the possible effects of novel virulence factors such as OMVs in development of *H. pylori*-associated extragastroduodenal diseases.

*Helicobacter pylori*-released OMVs are abundant from outer membrane proteins (OMPs), toxins, several adhesins, and different molecules known to stimuli cell proliferation, chemokine and cytokine production, and other host cellular responses [30]. In our previous work, we demonstrated that *H. pylori*-derived OMVs may alter the content of hepatocyte-originated exosomes, and such modified exosomes could possibly activate HSCs and promote liver fibrosis progression. We reported that hepatocytes extracted exosomes treated with *H. pylori*-derived OMVs can cause HSC activation and elevate the expression of hepatic fibrosis markers (*β*-catenin, vimentin, *α*-SMA, and TIMP-1) [31].

The current in vitro study showed that treatment of *H. pylori* OMVs regardless to isolate genotypes can act as a virulence attribute to active HSCs and induce liver fibrosis markers. Similar to our findings, there are several reports supporting the putative role of *H. pylori-*isolated OMVs in bacterial pathogenesis and disease progression. For instance, data obtained from an in vitro study by Hock et al. [32] indicated that *H. pylori* OMVs can strongly induce expression of COX-2 by the peripheral blood mononuclear cells (PBMC) and significantly elevate levels of PGE2 and IL-10, leading to suppression of human T cell responses. They also demonstrated that these effects were independent of VacA cytotoxin expression. Additionally, Choi et al. [33] investigated the clinical importance of *H. pylori* OMVs on the development of gastric cancer. They purified *H. pylori* OMVs from gastric secretion samples of patients with different gastric diseases including patients suffering from gastric and duodenal ulcer and gastric cancer. Their study revealed that *H. pylori*-derived EVs, which are rich in the gastric secretions of GC patients, are involved in the induction of inflammatory response and cancer progression in the stomach. Another study conducted by Zhang et al. [34], demonstrated that *H. pylori* OMV packages can provoke the production of IL-8 in AGS cell line due to the presence of small noncoding RNAs (sR-2509025 and sR-989262) resulting in evasion of host immune surveillance and responses. These findings imply that *H. pylori*-derived OMVs are crucial factors, which can carry various pathogenic cargos, and play a key role in *H. pylori*-related diseases and malignancy, in particular extragastroduodenal disorders.

In the current study, according to proteomic data, we found 112 different proteins within the OMVs derived from four *H. pylori* virulent strains during the exponential phase of growth, which were abundant in OMPs and enzymes like catalase and urease. However, interestingly, our proteomics results confirmed that no CagA and VacA proteins were present in the extracted OMVs. Comparing to our findings, Mullaney et al. [30] showed the existence of VacA and CagA in *H. pylori* OMVs. They identified 162 OMV-associated proteins in the *H. pylori* strains J99 and 91 in the strain 11637. These reports showed that CagA and VacA proteins could be present or absent in OMVs derived from different *H. pylori* strains. Despite the fact that the CagA and VacA are known as major virulence factors in *H. pylori* disease development, presence of other potent pathogenic factors in OMVs may result in activation of HSCs. Furthermore, a previous study that was conducted by Carlsohn et al. [35] reported more than 60 unique membrane or membrane-associated proteins in different clinical isolates of *H. pylori*. Some membrane proteins such as BabA and Omp11 were detected to be expressed by 15 different clinical isolates of *H. pylori*. Also, they established a mass spectrometry-based method that allows detection of OMPs in clinical isolates of *H. pylori*. However, recently, it was also indicated that OMV contents are highly associated with the *H. pylori* OMV size range. The proteomic studies showed that adhesins (Hop, SabA, and BabA) are more abundant among larger OMVs compared to smaller OMVs, while smaller OMVs contain significantly more metabolic proteins. Interestingly, *H. pylori* survival- or virulence-related proteins such as neutrophil activating protein, VacA, urease A and B subunits, and the porin HopA were common in both small and large OMVs [5]. Moreover, the study conducted by Turkina et al. [36] identified that *H. pylori* OMVs containing CagA could interact with gastric epithelia and influence host gene transcription and potentially cause serious clinical outcomes as a consequence of *H. pylori* infection.

HSCs play an important role in the liver fibrosis process. Activated HSCs are identified by the overexpression of *α*-SMA and extreme expression of extra cellular matrix proteins (vimentin, *β*-catenin, and snail) through inflammatory cytokines such as TNF-*α*, IL-1*β*, and IL6, which increase the development of liver fibrosis [37, 38]. A study conducted by Camara et al. revealed that TNF-*α* markedly elevated the effects of TGF-*β*1 cytokine, a well-known profibrotic cytokine, on fibrosis [39]. Also, results obtained from Fielding et al. study demonstrated strictly the dependence of fibrosis on interleukin-6 (IL-6). Also, their study showed that repetitive inflammation process induced Th1 cell effector activation via IL-6 cytokine and increased STAT-1 (signal transducer and activator of transcription-1) pathway activation, which can cause fibrosis [40]. In this study, we showed overexpressing of *α*-SMA, vimentin, and *β*-catenin and downregulation of E-cadherin in HSCs treated with CagA and VacA-negative *H. pylori* OMVs. Our findings revealed the presence of 112 different proteins in the proteome of OMVs that potentially may engage in HSCs activation. Similar to our findings, Goo et al. [41] revealed that orogastric *H. pylori* infection could elevate the *α*-SMA mRNA expression in a liver fibrosis-induced murine model. It was also shown that the adverse effects of *H. pylori* on liver are not limited to cytotoxin producer strains. They demonstrated that not only the strain with no vacuolating cytotoxic activity such as SS1 strain with VacA genotype s2m2 but also the ATCC 43504 with VacA genotype s1m1 can induce liver fibrosis highlighting the role of other *H. pylori*-associated virulence attributes in the pathogenesis of liver fibrosis. However, Krzysiek-Maczka et al. [42] showed that VacA- and CagA-positive *H. pylori* strains could elevate the vimentin and *α*-SMA mRNA expression and cause differentiation of normal fibroblasts to cancer-associated fibroblasts (CAFs) using the normal rat gastric epithelial cells (RGM-1). Moreover, Yang et al. [43] in in vivo study demonstrated that *H. pylori* infection promotes gastric epithelial cells apoptosis through downregulation of E-cadherin and overexpression of cleaved caspase-3. Also, Yu et al. [44] showed that CagA is able to activate vimentin and TWIST1 expression and downregulates programmed cell death factor 4 (PDCD4), which inhibits E-cadherin expression. They concluded that *H. pylori* CagA protein may induce epithelial-mesenchymal transition (EMT) process in cancerous gastric cells. Their results provide new findings about *H. pylori* infection effects on molecular network of gastric cancer and presents a new signaling cascade of EMT induction in gastric cancer cell lines.

## 5. Conclusions

In recent years, the impact of *H. pylori* infection and its OMVs in development of extragastroduodenal diseases especially liver diseases have attracted great attention. However, because of insufficient documents in this regard, the virulence attributes and mechanisms behind *H. pylori* extragastroduodenal pathogenesis are not clear yet. In summary, the current study revealed the potential role of *H. pylori*-derived OMVs in HSC activation. Also, we showed that CagA- and VacA-negative OMVs could trigger activation of HSCs. Interestingly, we found that OMVs from more virulent *H. pylori* strains (OMVs extracted from strains 1, 2, 4, and 5) could have more potent impacts on induction of liver fibrosis compared to less virulent strains. Further research and in vivo studies are required to elucidate the precise role of *H. pylori*-derived OMVs in stimulation of liver fibrosis and to provide further understanding of the putative mechanisms engaged in *H. pylori* liver-associated disorders.

## Figures and Tables

**Table 1:** Clinical *H. pylori* strains used in this study.

Strain no.	CagA	CagL	VacA	BabA2	SabA	OipA	CagPAI	Disease outcome	Age	Gender
1	+	+	s1m2	+	+	On	Partial	PUD	49	M
2	+	+	s1m1	+	—	On	Intact	CG	54	M
3	—	—	s2m2	+	+	Off	Completely deleted	CG	43	F
4	+	+	s1m1	+	+	On	Intact	PUD	25	F
5	+	+	s1m2	+	+	On	Intact	PUD	60	F

PUD: peptic ulcer disease; CG: chronic gastritis; male (M), female (F). Strain number 3 was used as a less virulent strain.

**Table 2:** Quantification proteomics analysis of OMVs derived from pathogenic clinical strains of *H. pylori*.

Protein ID	Protein description	LFQ intensity OMV 1	LFQ intensity OMV 2	LFQ intensity OMV 4	LFQ intensity OMV 5	Possible function
*Outer membrane proteins (OMP)*						
WP_000595790.1	Outer membrane beta-barrel protein	504540	3604860	963000	4892000	OMP/colonization/adhesion
WP_000768579.1	Outer membrane beta-barrel protein HofC	0	203850	0	184520	OMP/colonization/adhesion
WP_000395375.1	Hop family outer membrane protein (OMP) HopE	0	245410	693600	304390	OMP/colonization/adhesion/type 4 secretion system assembly
WP_000753173.1	Hop family adhesion BabB (Omp19)	0	0	0	73925	Blood group antigen binding adhesin B (BabB)
WP_000592437.1	Hop family adhesion AlpA (Omp20)	0	0	0	1082800	Adherence-associated lipoprotein A (AlpA)
WP_000812546.1	Hop family adhesion AlpB (Omp21)	0	0	0	277750	Adherence-associated lipoprotein B (AlpB)
WP_000831156.1	Outer membrane protein Omp18	0	1459100	0	429570	OMP/immune stimulator
WP_000751514.1	Hop family adhesion HopQ (Omp27)	0	362580	0	0	OMP27/CagA transporter
WP_000716249.1	Hop family adhesion BabA (Omp28)	0	0	1279040	1267000	Blood group antigen binding adhesin A (BabA)

*Protein with unknown functions*						
WP_001268661.1	Hypothetical protein	0	0	0	50133	Unknown
WP_000725112.1	Hypothetical protein	0	249580	0	196290	Unknown
WP_000446639.1	DUF874 family protein	0	29293	0	0	Unknown
WP_001225999.1	Hypothetical protein	23272	0	0	0	Unknown
WP_001228436.1	Hypothetical protein	0	0	0	189012	Unknown
WP_001202825.1	Hypothetical protein	0	0	0	80665	Unknown
WP_000323695.1	DUF3944 domain-containing protein	0	450110	154830	561010	Unknown

*Antibiotic resistance*						
WP_000912892.1	Leucyl aminopeptidase	24719300	39469000	40399000	40792000	Colonization/metronidazole resistant
WP_000670110.1	NAD(P)H-dependent oxidoreductase	0	0	194590	0	Metronidazole resistant

*Colonization factors*						
WP_000738964.1	Polyisoprenoid-binding protein	0	0	0	1887810	Colonization
WP_001174648.1	Thiol peroxidase	0	0	0	147870	Stress resistance and host colonization
WP_001285084.1	Acetone carboxylase subunit alpha	10039200	2686780	8418600	17540800	Colonization
WP_001862402.1	Acetone carboxylase subunit gamma	0	0	1948520	0	Colonization
WP_000731297.1	Fibronectin type III domain-containing protein	0	229900	0	175690	Colonization/type 3 secretion system
WP_001269995.1	Tol/Pal (colicin-tolerant/peptidoglycan-associated lipoprotein) system protein TolB	0	0	0	53991	TolB
WP_000795978.1	LPP20 lipoprotein	2583170	3121670	4231600	5133700	Colonization
WP_000961643.1	Peroxiredoxin	21375000	20724500	16015000	24932200	Colonization
WP_001215729.1	Aliphatic amidase	1222630	176590	4558450	7925500	Ammonia production/increase colonization
WP_001254471.1	Gamma-glutamyltransferase	0	0	0	48692	Gastric colonization

*Acid resistance*						
WP_000038087.1	Nickel-dependent hydrogenase large subunit	0	422490	0	311320	Urease maturation protein
WP_000709479.1	Polyisoprenoid-binding protein	0	0	353520	0	Acid response protein
WP_000779223.1	Urease subunit alpha	48115000	112113000	321814000	107958000	Urease A
WP_000724295.1	Urease subunit beta	87129000	68324000	274879000	157816000	Urease B
WP_001099471.1	Urease accessory protein UreD	0	761510	988360	334590	Urease D

*Potent virulence factors*						
WP_000846523.1	YbhB/YbcL family Raf kinase inhibitor-like protein	0	0	199250	0	Induce tumorigenesis
WP_001040419.1	Polysaccharide deacetylase	685340	1377940	0	2623860	PG amidase/immune response inducer
WP_001053815.1	Acetyl-CoA carboxylase biotin carboxyl carrier protein	0	0	0	44002	Adenocarcinoma progress
WP_000461024.1	ATP-dependent protease subunit HslV	0	0	0	32755	Protease activity
WP_000890837.1	Tumor necrosis factor alpha-inducing protein	0	1332180	0	319850	Accelerate inflammation and cancer development
WP_000467802.1	Methyl-accepting chemotaxis protein	2200500	4482860	3225630	3047290	Chemotaxis
	Periplasmic serine endoprotease DegP-like	0	657760	909990	770250	Protease high temperature requirement A (HtrA)
WP_000034151.1	Acyl-ACP--UDP-N-acetylglucosamine O-acyltransferase	0	93066	0	0	LPS assembly
WP_000846461.1	DNA starvation/stationary phase protection protein	7560800	6572800	16870200	11009900	Accumulation of neutrophils and monocytes at the site of infection
WP_000858181.1	3-Deoxy-8-phosphooctulonate synthase	0	50028	0	0	3-Deoxy-d-manno-octulosonic acid (KDO)
WP_000739470.1	Peptidylprolyl isomerase CBF2	0	0	0	358410	TH17 inflammation inducer
WP_000540573.1	ATP-dependent Clp endopeptidase proteolytic subunit ClpP	0	264590	0	563180	ATP-dependent caseinolytic proteases (ClpP)
*Metabolism and vital functions*						
WP_000506216.1	Enoyl-ACP reductase FabI	112930	0	0	0	Lipid metabolism
WP_000866624.1	Peptide-methionine (R)-S-oxide reductase MsrB	0	0	1182160	0	Repair enzyme for proteins that have been inactivated by oxidation
WP_000422563.1	DEAD/DEAH box helicase	0	0	0	45347	Helicase
WP_001207734.1	Ketol-acid reductoisomerase	0	1747020	1312860	186410	Amino acid synthesis enzyme
WP_001204347.1	Menaquinone biosynthesis decarboxylase	0	0	0	33451	Respiratory mechanism
WP_000662799.1	M3 family oligoendopeptidase	214060	52823	759950	0	Peptidoglycan endopeptidase
WP_000524601.1	Redox-regulated ATPase YchF	0	84152	0	0	Regulator protein
WP_000608576.1	2,3,4,5-Tetrahydropyridine-2,6-dicarboxylate N-succinyltransferase	0	173380	0	0	Amino acid synthesis enzyme/unknown function
WP_001006979.1	Acetyl-CoA C-acetyltransferase	463830	0	0	0	Metabolic enzyme
WP_000650637.1	Hydantoinase/oxoprolinase family protein	9456000	3187340	7925600	17515000	Metabolic enzyme
WP_000862222.1	GMP reductase	0	0	32786	0	Nucleic acid catabolic enzyme
WP_001268933.1	ADP-glyceromanno-heptose 6-epimerase	0	0	0	227002	Catabolic enzyme
WP_001126584.1	Carbamoyl-phosphate synthase large subunit	0	0	402270	0	Nitrogen metabolism
WP_001159546.1	4-Hydroxy-tetrahydrodipicolinate synthase	0	0	0	370800	Metabolic enzyme
WP_000060246.1	AAA family ATPase	0	0	0	612130	ATPases
WP_000062669.1	3-Methyl-2-oxobutanoate hydroxymethyltransferase	246880	1145910	0	559400	Metabolic enzyme
WP_000037869.1	DNA-directed RNA polymerase subunit beta/beta	464440	1495780	0	1015630	RNA polymerase
WP_000534771.1	Formamidase	0	0	0	856430	Nitrogen metabolism
WP_001160526.1	Class II fumarate hydratase	0	0	1094120	0	Nitrogen metabolism
WP_001862586.1	S41 family peptidase	0	0	565030	0	Endopeptidase
WP_000438056.1	3-Hydroxyacyl-ACP dehydratase FabZ	0	0	97338	0	Catabolic enzyme
WP_000780126.1	UbiX family flavin prenyltransferase	0	127650	0	256960	Catabolic enzyme
WP_000616334.1	Transcription-repair coupling factor	0	0	0	310300	Repair enzyme
WP_000963128.1	Recombinase RecA	170880	0	0	0	RecA
	Superoxide dismutase [Fe]	0	0	0	749400	Superoxide dismutase
WP_000955673.1	Phosphopyruvate hydratase	0	56407	0	0	Enolase
WP_000412947.1	Peroxiredoxin	1152900	0	0	0	Catabolic enzyme
WP_000080506.1	F0F1 ATP synthase subunit alpha	6236520	12373400	3033330	3668760	ATPase
WP_001863096.1	F0F1 ATP synthase subunit beta	1873140	2158280	2582800	1605730	ATPase
WP_000289187.1	NADP-specific glutamate dehydrogenase	0	496220	8149800	2938760	Metabolic enzyme
WP_000864547.1	DNA-directed RNA polymerase subunit alpha	0	0	0	22302	RNA polymerase
WP_001040579.1	Elongation factor Tu	2547840	2607030	1626200	1537060	Elongation factor
WP_000117507.1	Citrate synthase	2217840	4356700	3076740	2120940	Metabolic enzyme
WP_000323988.1	Isocitrate dehydrogenase (NADP(+))	0	555280	0	331560	Metabolic enzyme
WP_001242837.1	Cystathionine gamma-synthase	0	0	873810	0	Metabolic enzyme
WP_000476591.1	Porphobilinogen synthase	0	0	0	74893	Metabolic enzyme
WP_000002194.1	F0F1 ATP synthase subunit gamma	0	0	65433	0	ATPase
WP_001221712.1	IMP dehydrogenase	806320	1544980	0	1451060	Metabolic enzyme
WP_001124018.1	Phosphogluconate dehydratase	158780	161430	197580	608640	Metabolic enzyme
WP_001217520.1	Aspartate ammonia-lyase	1172670	826180	924680	303884	Nitrogen metabolism
WP_000131629.1	Ribonuclease J	0	0	0	127260	Ribonuclease
WP_000774319.1	Excinuclease ABC subunit (UvrC)	0	158820	0	0	UvrC
WP_000564420.1	Thioredoxin-disulfide reductase	0	0	304110	0	Metabolic enzyme
WP_000187711.1	Purine-nucleoside phosphorylase	0	0	0	233580	Metabolic enzyme
WP_010875529.1	Excinuclease ABC subunit UvrA	0	121980	0	0	UvrA
WP_000020199.1	Thioredoxin	0	0	918110	1172960	Metabolic enzyme
WP_000247370.1	Catalase	39582000	69503000	262561000	94226000	Catalase
WP_000637150.1	Type I glutamate--ammonia ligase	0	0	3351300	0	Metabolic enzyme
WP_000053246.1	Glutamate--tRNA ligase	0	0	0	154360	Metabolic enzyme
WP_000699284.1	Type II 3-dehydroquinate dehydratase	0	4075540	0	8030600	Catabolic enzyme
*Motility*						
WP_000885496.1	Flagellin A	798030	3071800	50265700	2449870	Flagellin A
WP_000010001.1	Flagellin B	0	132380	0	0	Flagellin B
WP_000646667.1	Flagellar sheath lipoprotein HpaA	0	766340	0	1061070	N-Acetylneuraminyllactose-binding hemagglutinin

*Ribosomal proteins*						
WP_001018245.1	50S ribosomal protein L7/L12	3165000	0	0	0	L7/L12
WP_000529962.1	30S ribosomal protein S3	0	0	134180	0	S3
WP_001862443.1	50S ribosomal protein L9	0	0	111940	0	L9

*Iron-related proteins*						
WP_000934548.1	HugZ family heme oxygenase	0	0	3413400	0	Iron acquisition
WP_000949202.1	Nonheme ferritin	5908500	9860700	9396400	14801600	Ferritin like protein

*Others*						
WP_000342347.1	Hybrid sensor histidine kinase/response regulator	0	192370	0	0	Response protein
WP_000785752.1	Cag pathogenicity island protein Cag1	0	203798	0	0	Membrane apparatus of T4SS
WP_000714010.1	Insulinase family protein	0	0	0	105420	Insulinase/unknown activity
WP_001265981.1	NAD(P)-dependent alcohol dehydrogenase	0	0	0	439940	Sensor protein
WP_000855958.1	Transporter substrate-binding domain-containing protein	2392480	3797000	4192600	2420660	Transporter
WP_029671489.1	Outer membrane beta-barrel protein	0	0	0	64626	Transporter
WP_001862559.1	ABC transporter substrate-binding protein	0	0	0	948250	Transporter
WP_001210849.1	Pyridoxine 5-phosphate synthase	3573670	8087900	1476140	2574090	Transferase
WP_000671934.1	Cochaperone GroES	2343300	3182200	2711900	3589100	Chaperone
WP_001040293.1	Chaperonin GroEL	64544000	136783000	55262000	109558000	Chaperone
WP_000664941.1	Copper-translocating P-type ATPase CopA	781570	0	0	0	Chemotaxis/heat-shock response

OMV1: OMVs derived from *H. pylori* strain 1; OMV2: OMVs derived from *H. pylori* strain 2; OMV4: OMVs derived from *H. pylori* strain 4, OMV5: OMVs derived from *H. pylori* strain 5. Abbreviations: *H. pylori* outer membrane protein family C: HofC, *Helicobacter* outer membrane proteins: Hop, nicotinamide adenine dinucleotide: NAD, Lpp20: lipoprotein 20, heat-shock locus V: hslV, lipopolysaccharides: LPS, protease high temperature requirement A: HtrA, deoxyribonucleic acid: DNA, 3-deoxy-d-manno-octulosonic acid: KDO, cell binding factor 2: CBF2, ATP-dependent caseinolytic proteases: ClpP, fatty acid biosynthesis I: FabI, methionine sulfoxide reductase B: MsrB, guanosine monophosphate: GMP, adenosine diphosphate: ADP, ATPases associated with diverse cellular activities: AAA, adenosine triphosphatase: ATPases, recombinase A: RecA, nicotinamide adenine dinucleotide phosphate: NADP, thermal unstable: Tu, inosine monophosphate: IMP, ultraviolet radiation: Uvr, *Helicobacter pylori* adhesion A: HpaA, heme utilization gene: Hug, cytotoxic associated gene: cag, copper-translocating P-type ATPase: CopA.

## Data Availability

Data generated during the study are available from the corresponding authors by request.

## Supplementary Materials

Supplementary 1 Primers used for real-time PCR assay in this study.

Supplementary 2 Proteomics analysis of each OMV derived from *H. pylori* strains used in this study.
