# Supplementary material for: The Effects of Helicobacter pylori-Derived Outer Membrane Vesicles on Hepatic Stellate Cell Activation and Liver Fibrosis In Vitro
Source: Biomed Res Int. 2023 Apr 12;2023:4848643. doi: 10.1155/2023/4848643 (PMC10116224; doi:10.1155/2023/4848643)
Supplement: Supplementary 1 — Primers used for real-time PCR assay in this study. [file 4848643.f1.docx]

**Table S1.** Primers used for real-time PCR assay in this study.

| **Gene** | **Sequences** | **Product size (bp)** |
| --- | --- | --- |
| β-catenin | F-GGGTAGGGTAAATCAGTAAGAGGT  R-GCATCGTATCACAGCAGGTT | 261 |
| E-cadherin | F-TGCTCTTGCTGTTTCTTCGG  R-CTTCTCCGCCTCCTTCTTC | 280 |
| Snail | F-CACTATGCCGCGCTCTTTC  R-TCCTGGAAGGTAAACTCTGGAT | 310 |
| Vimentin | F-CCAGGCAAAGCAGGAGTC  R-CGAAGGTGACGAGCCATT | 426 |
| α-SMA | F-AGACGGGAATCCTGTGAAGC  R-TGTCCCATTCCCACCATCAC | 314 |
| GAPDH | F-GAAGGTGAAGGTCGGAGTCA  R-AATGAAGGGGTCATTGATCA | 245 |
