# Supplementary material for: The Effects of Helicobacter pylori-Derived Outer Membrane Vesicles on Hepatic Stellate Cell Activation and Liver Fibrosis In Vitro
Source: Biomed Res Int. 2023 Apr 12;2023:4848643. doi: 10.1155/2023/4848643 (PMC10116224; doi:10.1155/2023/4848643)
Supplement: Supplementary 2 — Proteomics analysis of each OMV derived from H. pylori strains used in this study. [file 4848643.f2.docx]

**Table S2.** Proteomics analysis of each OMV derived from *H. pylori* strains used in this study.

| **OMV 1** | **OMV 2** | **OMV 4** | **OMV 5** | **Common factors** |
| --- | --- | --- | --- | --- |
| enoyl-ACP reductase FabI | YchF | hypothetical protein | YbhB/YbcL family Raf kinase inhibitor-like protein | aliphatic amidase |
| hypothetical protein (unknown) | hybrid sensor histidine kinase/response regulator | helicase | peptide-methionine (R)-S-oxide reductase MsrB | polysaccharide deacetylase |
| acetyl-CoA C-acetyltransferase | Cag1 | polyisoprenoid-binding protein | HugZ family heme oxygenase | ketol-acid reductoisomerase |
| RecA | DUF874 family protein | acetyl-CoA carboxylase biotin carboxyl carrier protein | acetone carboxylase subunit gamma | M3 family oligoendopeptidase |
| CopA | 2,3,4,5-tetrahydropyridine-2,6-dicarboxylate N-succinyltransferase | thiol peroxidase | GMP reductase | Omp11 |
| CusA/CzcA family heavy metal efflux RND transporter | Hop family adhesin HopQ | menaquinone biosynthesis decarboxylase | carbamoyl-phosphate synthase large subunit | outer membrane beta-barrel protein HofC |
| NadA | acyl-ACP--UDP-N-acetylglucosamine O-acyltransferase | ATP-dependent protease subunit HslV | NAD(P)H-dependent oxidoreductase | leucyl aminopeptidase |
| L7/L12 | phosphopyruvate hydratase | insulinase family protein | polyisoprenoid-binding protein | tumor necrosis factor alpha-inducing protein |
|  | 3-deoxy-8-phosphooctulonate synthase | hypothetical protein | class II fumarate hydratase | methyl-accepting chemotaxis protein |
|  | excinuclease ABC subunit UvrC | ADP-glyceromanno-heptose 6-epimerase | S41 family peptidase | nickel-dependent hydrogenase large subunit |
|  | excinuclease ABC subunit UvrA | BabB | 3-hydroxyacyl-ACP dehydratase FabZ | HyuA |
|  | flagellin B | AlpA | S3 | acetone carboxylase subunit alpha |
|  | CheAY2/CheF | AlpB | L9 | Omp15 |
|  |  | 1 4-hydroxy-tetrahydrodipicolinate synthase | cystathionine gamma-synthase | fibronectin type III domain-containing protein |
|  |  | ATPase | F0F1 ATP synthase subunit gamma | HtrA |
|  |  | NAD(P)-dependent alcohol dehydrogenase | thioredoxin-disulfide reductase | 3-methyl-2-oxobutanoate hydroxymethyltransferase |
|  |  | gamma-glutamyltransferase | type I glutamate--ammonia ligase | Omp18 |
|  |  | TolB |  | transporter substrate-binding domain-containing protein |
|  |  | formamidase |  | RNA pol |
|  |  | outer membrane beta-barrel protein |  | BabA |
|  |  | ABC transporter substrate-binding protein |  | UbiX family flavin prenyltransferase |
|  |  | SOD |  | transcription-repair coupling factor |
|  |  | RNA pol |  | pyridoxine 5-phosphate synthase |
|  |  | porphobilinogen synthase |  | DUF3944 domain-containing protein |
|  |  | peptidylprolyl isomerase CBF2 |  | co-chaperone GroES and GroEL |
|  |  | ribonuclease |  | flagellin A |
|  |  | purine-nucleoside phosphorylase |  | LPP20 lipoprotein |
|  |  | glutamate-tRNA |  | urease A |
|  |  | GatB |  | peroxiredoxin |
|  |  | ClpX |  | DNA starvation/stationary phase protection protein |
|  |  |  |  | non-heme ferritin |
|  |  |  |  | flagellar sheath lipoprotein HpaA |
|  |  |  |  | peroxiredoxin |
|  |  |  |  | F0F1 ATP synthase subunit alpha and beta |
|  |  |  |  | NADP-specific glutamate dehydrogenase |
|  |  |  |  | elongation factor Tu |
|  |  |  |  | citrate synthase |
|  |  |  |  | isocitrate dehydrogenase (NADP(+)) |
|  |  |  |  | IMP dehydrogenase |
|  |  |  |  | phosphogluconate dehydratase |
|  |  |  |  | aspartate ammonia-lyase |
|  |  |  |  | ClpP |
|  |  |  |  | thioredoxin |
|  |  |  |  | urease B |
|  |  |  |  | catalase |
|  |  |  |  | urease D |
|  |  |  |  | type II 3-dehydroquinate dehydratase |
